# Living systematic reviews in rehabilitation science can improve evidence-based healthcare

**DOI:** 10.1186/s13643-021-01857-5

**Published:** 2021-12-07

**Authors:** S. Elbers, H. Wittink, U. Kaiser, J. Kleijnen, J. Pool, A. Köke, R. Smeets

**Affiliations:** 1 Research group Lifestyle & Health, Research Centre Healthy and Sustainable Living, University of Applied Sciences Utrecht, P.O. Box 12011, 3508 AA Utrecht, The Netherlands; 2 Department of Rehabilitation Medicine, Research School CAPHRI, Faculty of Health, Life Sciences and Medicine, Maastricht University, Maastricht, The Netherlands; 3 Comprehensive Pain Center, Medical Faculty Technical University Dresden, Dresden, Germany; 4 University Hospital Carl Gustav Carus Dresden, Dresden, Germany; 5 Department of Family Medicine, Research School CAPHRI, Faculty of Health, Life Sciences and Medicine, Maastricht University, Maastricht, The Netherlands; 6 Centre of Expertise in Pain and Rehabilitation, Adelante, Maastricht, The Netherlands; 7 South University of Applied Sciences Heerlen, Heerlen, The Netherlands; 8 CIR Revalidatie, location Eindhoven, Eindhoven, The Netherlands; 9 Pain in Motion International Research Group (PiM), https://www.paininmotion.be

**Keywords:** Systematic review, Living systematic review, Rehabilitation, Chronic pain, Methods, Meta-analysis

## Abstract

Although systematic reviews are considered as central components in evidence-based practice, they currently face an important challenge to keep up with the exponential publication rate of clinical trials. After initial publication, only a minority of the systematic reviews are updated, and it often takes multiple years before these results become accessible. Consequently, many systematic reviews are not up to date, thereby increasing the time-gap between research findings and clinical practice. A potential solution is offered by a living systematic reviews approach. These types of studies are characterized by a workflow of continuous updates which decreases the time it takes to disseminate new findings. Although living systematic reviews are specifically designed to continuously synthesize new evidence in rapidly emerging topics, they have also considerable potential in slower developing domains, such as rehabilitation science. In this commentary, we outline the rationale and required steps to transition a regular systematic review into a living systematic review. We also propose a workflow that is designed for rehabilitation science.

## Background

Systematic reviews are considered to be the foundations of evidence-based practice [1–3]. The structured method of identifying, appraising, evaluating and synthesizing primary research findings facilitates clinical decision making based on the available evidence at a given time [4, 5]. Despite its widespread success, researchers have identified several problems in the conduct of systematic reviews. First, the average duration of performing a systematic review is almost a year from initial search to publication [1]. Furthermore, only a minority of the published reviews are updated and the median estimated time to update is approximately 3 years for Cochrane reviews and 5 years for non-Cochrane reviews [6, 7]. Given the continuously increasing flow of new trials, many systematic reviews become rapidly out of date [4, 8]. Second, poorly prepared systematic reviews are susceptible to bias, such as incomplete search strategies [9], errors in data extraction [10], and inter-rater disagreement in risk of bias assessments [11]. Although the effect on outcomes may be limited [12], the current system does not easily allow for corrections or updated practices after publication of the initial results.

Living systematic reviews (LSRs) are proposed as an alternative method to traditional systematic reviews that may offer a solution to these challenges. A systematic review is considered “living” when it includes a system by which it regularly incorporates newly available evidence into the analysis [13]. Generally, this system consists of a predefined cyclic workflow with planned updates at intervals ranging from days to months. Assisted by the availability of recent software developments that provide (semi-)assisted solutions for the review process, these updates take only a fraction of the time of conducting a baseline review [14, 15]. The chosen timeframe is at the discretion of the researchers and generally depends on the speed at which a topic of interest is expected to develop. Although LSRs have specifically been designed for rapid updating cycles of emerging topics, such as mapping the COVID-19 trials (COVID-NMA), protocols have been published that include updating cycles of 12 months [16–18].

The aim of this commentary is to illustrate the process of transitioning a regular review into a LSR and to propose a workflow for updating LSRs over time. We will use a recently performed research project where we transitioned a regular systematic review into a LSR as an example to discuss the required steps and key issues.

## Main text

### Characteristics of the baseline review

We performed the initial review in September 2020 [19] to identify the change in outcomes over time and the between-study heterogeneity for interdisciplinary pain treatment programs for patients with chronic pain. All longitudinal study designs such as case series and RCTs were considered. Our main reason for including more study types than RCTs was that carefully controlled inclusion criteria and treatment procedures do often not reflect the heterogeneous reality of clinical practice in the domain of rehabilitation [20–23]. In addition, many treatment programs disseminate their results through case series, which contain important information regarding the change over time. The abstract screening, full-text selection, data extraction, and risk of bias assessment was performed in duplicate. Primary studies were eligible if they included interdisciplinary multimodal pain treatment (IMPT) programs as intervention for patients with chronic primary pain that was primarily perceived in musculoskeletal structures. Furthermore, studies had to include a baseline and a follow-up assessment of at minimum 12 months post treatment for at least one of the outcomes of interest: Physical function, pain interference, self-efficacy, depression, anxiety, anger, general emotional function, social role functioning, and pain intensity. We assessed risk of bias with the JBI critical appraisal checklist for case series. Our data analysis included the change over time per outcome, by calculating standardized mean differences between pre-, post-, and final follow-up timepoints.

### LSR protocol

A LSR starts with a baseline systematic review that is in line with the PRISMA reporting standard [13, 24]. In addition, there should be a study protocol that includes a rationale for maintaining a LSR as well as planned methodological and statistical approaches that are in line with multiple iterations over time [5, 13].

When the decision was made for the transition of this systematic review into a living systematic review, we submitted a new study protocol in PROSPERO (CRD42021247142). We chose to create a new record instead of updating the record of the baseline review, because the workflow and iterative nature of the living review are substantially different. The main rationale for this living systematic review was to provide and maintain an up-to-date overview of all interdisciplinary pain treatment programs that include a baseline and a follow-up measurement of at least 12 months post treatment on one of our outcomes of interest. Other reasons included a faster inclusion of primary studies in a meta-analysis, the possibility of accommodating new methodological developments into the study, and to update data extraction forms at a later stage, to include follow-up studies or correct omissions or errors.

### Workflow

LSRs require long-term commitment of a research team, which can lead to a substantial ongoing workload [25]. Detailed planning of a feasible workflow that allows for updates of the procedures over time is therefore recommended [24, 26]. Researchers should anticipate that the research team is likely to change over time, and include training programs to calibrate assessment practices, and to ensure a correct interpretation of the screening and data extraction forms. This is also true for leaving the possibility open to integrate new digital developments to (semi-)assisted approaches within the review process at a later stage [13, 15].

**Fig. 1:**
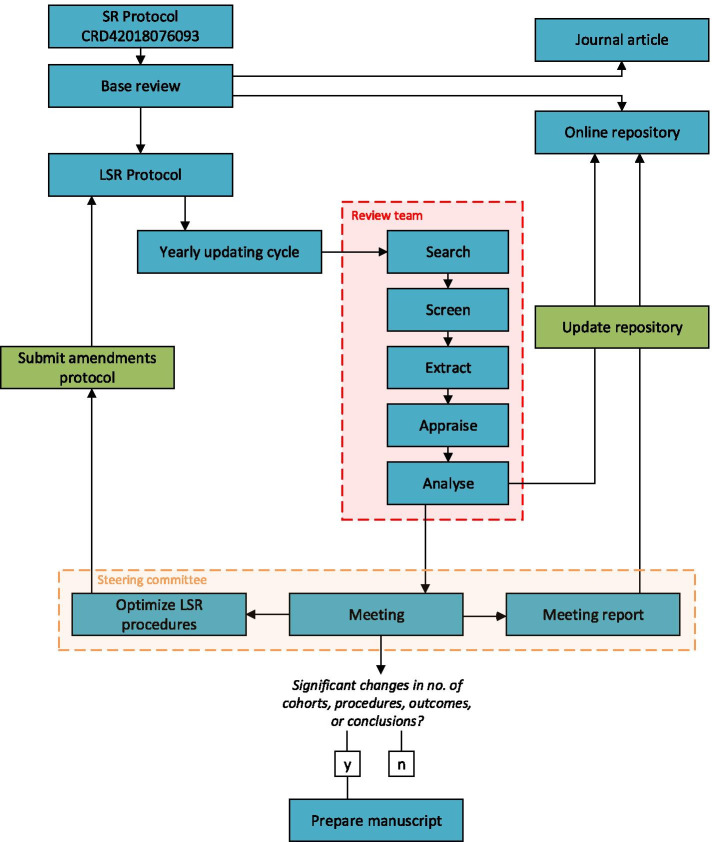
Workflow diagram of the living systematic review

Figure 1 depicts the workflow for the current project. Each year, we will rerun the search in all databases. New records will be imported into Endnote and deduplicated using the procedure of Bramer and colleagues [27]. The set of unique records will then be imported into web application Rayyan, for masked screening in duplicate [28]. Potential eligible studies will be imported into Endnote to help find the full text versions for these references. We will use Google Forms to perform the second-round selection, data extraction and risk of bias assessment. The advantage of these digital forms is that they are stored in an online spreadsheet that is connected to our digital application. Hence, new studies are automatically integrated into the online tables and calculations. The results of the update will be evaluated by the steering committee during the yearly meeting.

The involved researchers are divided over two teams. Members of the steering committee will meet on a yearly base and decide whether new results justify a peer review publication, based on the number of new cohorts, changes in outcomes or conclusions. At minimum, we will publish an update every 3 years. This timeframe has been chosen to balance the prevention of redundant research efforts and scientific publications with the notion that an absence of any change also contains valuable information. The steering committee will also discuss potential methodology improvements or amendments to the procedures, including the opportunity to integrate large scale routinely collected data [14], the refinement of the data extraction form based on a newly developed checklist [29], and initiatives to increase participation of the research community [30]. Finally, the steering committee will evaluate the need for continuation of this LSR project based on the replication checklist by Tugwell and colleagues [31]. The meeting notes will be added as appendix to the online repository. Members of the review team will be responsible for screening, study selection, data extraction, and risk of bias assessment. They will receive training in all software applications and review procedures. For this purpose, we will create a training dataset consisting of 20 studies identified by the search of the baseline review, which will contain a mix of included and excluded studies. As recommended by Gagnier and colleagues [32], we aim to always include members with expertise in pain rehabilitation.

### Dissemination

In addition to journal articles, LSR results are often disseminated through online applications [30, 33]. These digital platforms include all extracted data as well as interactive functions to visualize or summarize (a subset of) the data that is specifically relevant to the end-user [4, 30, 33]. Consequently, these interactive functions provide the opportunity to supply information to various stakeholders, including researchers, clinicians, policy makers, and patients.

To disseminate the results in an interactive way, we developed an R Shiny application that provides relevant and most up to date information of this review project [34]. The online platform contains key tables, time series, and forest plots. To optimize transparency, the raw data extraction forms and risk of bias assessments are also made available. Using functionalities of Shiny software, all plots and figures include interactive functions, including tables with search, filter and sort functions, forest plots with adjustable corrections for within-subject correlations and time series, with cohort selection and export functions. These functions help users to navigate to the available data, to explore differences and similarities between cohorts and to perform sensitivity analyses. The source code is available through GitHub, which also registers the amendments that will be made over time [35]. To further increase the relevance for clinicians and policymakers, the platform could be expanded in future iterations with additional modules, such as GRADE recommendations, that improve the description, interpretation, and understanding of the dataset (Table 1).

**Table.1:** Steps for transitioning a systematic review into a living systematic revie

• Perform a baseline review	
• Develop a living systematic review protocol that includes a rationale for this type of study, a cyclic workflow, and a clear procedure for when to publish new results	
• Create a steering committee and review team. Anticipate changes in the team composition by documenting all previous decisions and developing training methods for the review	
• Design an online platform to report updated results. Consider all potential end-users when deciding on what information to disseminate and how to present this	

## Conclusions

Living systematic reviews provide a means to quickly integrate primary studies into meta-analyses, while upholding the scientific rigor of systematic reviews. Recent innovations in (semi-)assisted tools for conducting a systematic review have streamlined the procedures and have substantially reduced the efforts to update results over time. We believe that these up-to-date overviews, communicated through peer reviewed articles and online applications, positively impact the dissemination and implementation of scientific findings into clinical practice.

## Data Availability

The dataset generated by the living systematic review will be available in the impt-cohort repository: https://datascience.hu.nl/rsconnect/impt-cohorts/
